# The Vocal Repertoire of Adult and Neonate Giant Otters (*Pteronura brasiliensis*)

**DOI:** 10.1371/journal.pone.0112562

**Published:** 2014-11-12

**Authors:** Christina A. S. Mumm, Mirjam Knörnschild

**Affiliations:** 1 Institute of Experimental Ecology, University of Ulm, Ulm, Germany; 2 Institute of Experimental Ecology, Faculty of Natural Sciences, University of Ulm, Ulm, Germany; 3 Smithsonian Tropical Research Institute, Balboa, Panama; University of Windsor, Canada

## Abstract

Animals use vocalizations to exchange information about external events, their own physical or motivational state, or about individuality and social affiliation. Infant babbling can enhance the development of the full adult vocal repertoire by providing ample opportunity for practice. Giant otters are very social and frequently vocalizing animals. They live in highly cohesive groups, generally including a reproductive pair and their offspring born in different years. This basic social structure may vary in the degree of relatedness of the group members. Individuals engage in shared group activities and different social roles and thus, the social organization of giant otters provides a basis for complex and long-term individual relationships. We recorded and analysed the vocalizations of adult and neonate giant otters from wild and captive groups. We classified the adult vocalizations according to their acoustic structure, and described their main behavioural context. Additionally, we present the first description of vocalizations uttered in babbling bouts of new born giant otters. We expected to find 1) a sophisticated vocal repertoire that would reflect the species’ complex social organisation, 2) that giant otter vocalizations have a clear relationship between signal structure and function, and 3) that the vocal repertoire of new born giant otters would comprise age-specific vocalizations as well as precursors of the adult repertoire. We found a vocal repertoire with 22 distinct vocalization types produced by adults and 11 vocalization types within the babbling bouts of the neonates. A comparison within the otter subfamily suggests a relation between vocal and social complexity, with the giant otters being the socially and vocally most complex species.

## Introduction

The complexity of mammalian vocal repertoires is both the sum of several aspects of call structure and function, as well as of evolutionary and selective pressures acting on senders and receivers. Production and perception mechanisms, such as the anatomic structure of the vocal tract or auditory sensitivity, shape and limit the number of distinct vocalizations a species can produce and perceive [1]–[4]. As described by the source-filter theory [5], [6], source (i.e. larynx) induced acoustic parameters include duration, tempo, fundamental frequency and nonlinear phenomena [7], [8]. Nonlinear phenomena, being subharmonics, biphonation and deterministic chaos [8], seem to be a rather involuntary by-product of vocal production, nevertheless, increasing the diversity of acoustic signals [9]. Filter (i.e. supralaryngeal vocal tract) induced vocal parameters include formants which, among other cues, indicate the body size of the signaller [10].

Vocalizations can be internally referential, providing information on motivational state, individuality or social origin of the sender, or externally referential, providing information on external events for receivers [1]. According to Morton’s motivation-structural rules [11], the physical structure of mammalian and avian vocal signals reflects the motivation and context in which they are produced. Aggressive vocalizations should have lower frequencies and sound harsh, whereas friendly calls should have higher frequencies and sound pure [11]. Bradbury and Vehrencamp [12] refined these motivation-structural rules for the design of mate-attracting, courtship, territorial defence, threat and alarm signals.

The vocalizations within many mammalian repertoires are neither completely discrete, nor completely graded [13], [14]. They rather represent a combination of distinct calls, graded signals, variants, and transitions between them [12], [15], [16]. By varying certain acoustic parameters, animals can use variants of graded vocalizations to signal a more detailed information about their internal state, motivation, or the degree of external danger [12]. Segmental concatenation, being the combination of different vocal cues in one call type [17], as well as a structured combination of vocalizations, resulting in a different or more specific meaning than that of single calls, contribute to the enhancement of a vocal repertoire [18]–[20].

Cognitively advanced mammals such as primates do not only perceive and recognize distinct meaning from graded call subtypes [18], but may respond differently to apparently similar calls, or, conversely, may be able to derive the same meaning from structurally different vocalizations [21]. This shows that the acoustic structure of vocalizations can, but does not have to be linked with function [21]. Rather, communication is shaped by a combination of the information transmitted, the behavioural context, the relationship of the interacting individuals and, particularly in social groups, by the presence of bystanders [21], [22].

The ontogenetic development of a mammalian vocal repertoire undergoes different stages [23]. Some neonate or juvenile calls might disappear during development, whereas the number of vocalizations of the adult repertoire exceeds the number of juvenile calls [16], [23]. Structure, usage and the number of vocalizations may change until the full adult and species-specific repertoire is formed [16], [23], [24]. Typically, the fundamental frequency decreases with age and growth [16], [23]. The high frequency and pure tone quality of dependant offspring vocalizations are well suited to elicit parental care behaviour [11]. Babbling behaviour, generally described to facilitate practising the adult vocalizations [25]–[27], may also direct the attention of caregivers to the vocalizing cub [25], [26], [28].

Recently, the ‘social complexity hypothesis for communication’ [29] has been supported by several studies on non-human primates [30], [31], birds [32], [33], marmots [34] and carnivores [35], [36]. This hypothesis states that vocal complexity can be driven by social complexity [29]. Vocal complexity can thereby be classified in terms of vocal repertoire size [31], [34], [35], information content of the vocalizations or within the vocal system [32], as well as usage diversity of the vocal signals [30], [33]. Group size [32], [35], or the variety of social roles and relationships [34] can be used to measure social complexity [33]. Effects of habitat, predation pressure or species recognition do not necessarily promote vocal complexity even though they shape the structure of vocalizations [29]. Group size, as a measure for social complexity, is thought to favour the evolution of individual acoustic distinctiveness [37], as well as vocal complexity at the species level [30]. Living in social groups built of closely and frequently interacting individuals with different social roles is demanding and requires a sophisticated communication system (sensu [38], [39]). This allows the interacting individuals to deal adequately with challenges originating from their social structure.

With this paper, we want to push forward the formerly poorly noted otters, *Lutrinae*, into the general discussion of acoustic communication and vocal complexity. Generally, otters represent a well-suited taxon for testing the relation of sociality and vocal complexity (as suggested in [40]). They provide a wide variety of social organization from solitary living species to complex social groups [41] and the otter species can be differentiated by their vocalizations [42]. In this paper, we discuss social organization and vocal repertoire size as measures of complexity in otters.

Known for their conspicuous and frequently emitted vocalizations, giant otters (*Pteronura brasiliensis*) are interesting in terms of both their vocal repertoire and their complex social life. This species, endemic to lakes and rivers in Amazonian rainforests and wetlands [41], [43], is listed as ‘endangered’ in the red list of threatened species [44]. Giant otters usually live in family groups with the alpha-couple and their offspring from different years [42]–[45], resulting in group sizes between three to nine individuals [43], [44], [46]. A detailed study by Ribas [45] revealed a higher complexity of social organization and variety of relatedness in giant otter groups, differing from the general pattern of a ‘parent-brood model’ [45] described before. Subadult giant otters may stay as helpers within the family or leave their natal territory when reaching sexual maturity [42], [47], [48]. The dispersing individuals might stay solitary (‘transients’) [43], [46] or form temporary groups of unrelated members, until they establish their own territory and family group [45]. Group members show a strong association index [46], describing the probability of observing pairs of individuals together (Half-Weight Index, described in [49]). The group cooperates in breeding and territorial defence [43], [46], [50], [51], whereas it is still unclear, whether giant otters truly show cooperative hunting or not [43]. Mostly, group members fish in close proximity. Smaller sized fish are eaten individually, but elder group members might share fish with dependant offspring [41]–[43]. Giant otters feed together on larger prey like catfish or juvenile caimans ([43], own observation). Giant otter cubs join the fishing bouts of their family at an age of two months. Until they learn to fish on their own, they depend on food sharing from elder group members [43]. Starting with direct provision of small dead fish, elder group members will also provide larger or half-dead fish later on during development of the juveniles, to increase their abilities of capturing and handling prey ([43], own observation). Vocalizations of giant otters have partly been described by previous studies [42], [43], [52], [53]. Leuchtenberger [54] studied the vocal repertoire of wild giant otters in Brazil. She found 15 vocalization categories with several subtypes, resulting in a total of 19 distinct vocalizations. We recently reported vocal individuality in the cohesion calls of giant otters [40] and Leuchtenberger [54] showed sex differences in the giant otter alarm call ‘snort’.

To describe the vocal repertoire, we recorded and analysed air-borne and underwater calls of wild and captive giant otter groups and provide the behavioural context in which different vocalizations were produced. Furthermore, we described neonate vocalizations and contrast them with the adult vocal repertoire.

The aim of the present study was to give a detailed overview of the giant otters’ vocal repertoire and set it into the context of social complexity. Due to the outstandingly social system of giant otters within the *Lutrinae* (compare [41], [55]–[58]), we hypothesized to find a sophisticated vocal system with distinct vocalization types. Moreover, we hypothesized that giant otter vocalizations show a clear relationship between signal structure and signal function according to Morton’s motivation-structural rules, and that the neonate vocal repertoire would include precursors of the adult repertoire, as well as age-specific vocalizations.

## Materials and Methods

### Study sites and study animals

We recorded five wild and three captive giant otter groups. Group size varied from two to fifteen individuals, covering all age classes from new born cubs to adults (giant otter age classes according to [59]). We recorded the vocalizations of wild giant otters at five oxbow lakes, namely Cocha Cashu, Cocha Salvador (September to December 2011), Cocha Tres Chimbadas, Cocococha and Cocha Sandoval (April to July 2012) in the Cusco and Madre de Dios Departments, Peru. The lakes Cocha Cashu (N -11°53′3.9984″, E -71°24′28.0008″) and Cocha Salvador (N -11°59′45.9996″, E -71°13′59.0016″) are located within the Manu National Park. Cocha Sandoval (N -12°36′29.4336″, E -69°2′26.9988″) and Cocococha (N -12°49′0.624″, E -69°15′36.3456″) are within the Tambopata National Reserve and Cocha Tres Chimbadas (N -12°47′21.7932″, E -69°20′44.0988″) in the reserves buffer zone.

The recordings of the vocalizations of captive giant otters took place in three German zoos, Tierpark Hagenbeck (April 2009, May 2011 and July 2011), Zoo Duisburg (March 2011) and Zoo Dortmund (March 2009 and April 2011). The enclosures included separable indoor and outdoor areas. The indoor enclosures with artificial light mainly served as retreat areas for the animals and the outdoor enclosures with natural light were always open for visitors. When cubs were born, the zoos inhibited public access to the retreat areas. Giant otters were fed an amount of 2.0 to 6.0 kg of fish (trout, whiting and roach) per day and individual. The animals received supplementary fruit and vegetables as enrichment.

### Animal welfare

Since giant otters are listed as endangered by the IUCN red list of threatened species [44], we obtained official research permits (No. 014 S/C- 2011-SERNANP-PNM, 014-2012-SERNANP-JEF, 017-2012-SERNANP-JEF and 0167-2012-DGFFS-DGEFFS) for field work in Peru. The permits provided by SERNANP (Servicio Nacional de Áreas Naturales Protegidas), the Peruvian nature conservation authority and DGFFS (Dirección General Forestal y de Fauna Silvestre), the Peruvian agricultural department, allowed us to record wild giant otter groups. To habituate the giant otters to our presence, we never hid our activities, but kept a minimum observing distance of 10 m–50 m, depending on the giant otters’ activities. We increased the distance when new born cubs were present. Wild neonates were recorded coincidentally at one lake, as the group moved the cubs from one den to another.

In Germany, we received research permissions to conduct our study with the captive giant otters from the respective persons in charge (Tierpark Hagenbeck: veterinarian, Zoo Dortmund: zoo director and Zoo Duisburg: curator.). We never separated individual giant otters from the group during recording and abandoned recording when new cubs were born. At Tierpark Hagenbeck, an autonomous recording device was installed at the nesting box inside the indoor enclosure to record the neonates instead.

### Recording

We recorded air-borne vocalizations as wave files with a directional microphone (Sennheiser, MKH 416-P48U3) connected to a digital audio recorder (Zoom H2 Handy Recorder; 48 or 96 kHz sampling rate, 24 bit depth resolution). Underwater calls were recorded with a hydrophone (RESON TC4013 Hydrophone, 1 Hz to 170 kHz), amplified by a charge amplifier (#42003, P48), connected to a second audio recorder (Zoom H2 Handy Recorder; 96 kHz sampling rate, 24 bit depth resolution). We documented the behavioural context of air-borne vocalizations by spoken notes and video recordings (Sony, DCR SR-35 camcorder). Underwater behaviour and the behaviour of the captive neonate cubs inside the nesting box could not be recorded. In the wild, we followed the free ranging giant otters with one-person kayaks. The otters' daily activity period from sunrise to sunset (around 5 am to 5 pm), was monitored by two observers with four alternating three hours shifts. In the zoos, we conducted alternating three hours recording sessions, covering the giant otters’ activity period in the morning, afternoon or evening.

### Classification and analysis of the vocalizations

We classified the adult vocalizations acoustically and visually with respect to the behavioural context. Following Tyack and Miller [60], we named the distinct giant otter calls according to their structure and with reference to the classification by other authors. Subsequently, we assigned them to the behavioural context to avoid a confusion of call structure and function [60]. Only calls having a clear specific function were named accordingly (for instance the begging call and the contact call). Since the giant otter vocal repertoire includes several gradations and variations of the vocalizations [42], we restricted our analysis to vocalizations which could readily be distinguished by structure, context, or a combination of both.

We selected the calls for analysis from the original wave files in Raven Pro (version 1.4, Cornell University, Ithaca, New York). We restricted our selection to vocalizations with a good signal-to-noise ratio, which were not overlapped by other calls and for which we had additional information on the behavioural context for analyses. To reduce an effect of very similar or modulated vocalizations within calling bouts of the same individual [61], we selected the vocalizations from different recording dates whenever possible. Before measuring the acoustic parameters in Raven Pro, we conducted noise reduction (WavePad Sound Editor, version 4.52, NCH Software), erased bird calls or loud background noise (AviSoft SASlab Pro, version 5.1.23, Avisoft Bioacoustics, Berlin, Germany) and normalized the volume of the vocalizations (WavePad Sound Editor). In Raven Pro, we measured frequency variables from a spectrogram and a selection spectrum with high frequency resolution (Fast Fourier transform: 1024-point, window: Hann, overlap: 87.5%, temporal resolution: 2.67 ms, DFT size: 1024 samples, frequency resolution: 46.9 Hz) and time variables from the waveform. In sum, we measured 441 calls, stemming from 8 giant otter groups. Each group consisted of 6–15 individuals (totalling 66 individuals) but many vocalizations could not be attributed to a specific individual. Due to low sample size and low quality of the records, we did not measure the mating call. Neonate calls were not measured due to low recording quality, but are shown in the results to give an overview of the vocal repertoire of giant otters as accurately as possible. The vocalizations and their behavioural context were compared to former findings and descriptions by other authors.

### Measurements of acoustic parameters

Aiming for parameters that would best differentiate the vocalizations within the adult vocal repertoire, we analysed the calls both over their entire length and within four subunits of equal duration. Entire vocalizations were measured by selecting a spectrogram section from the highest to the lowest frequency visible (narrower when no harmonics were present and wider when harmonics were present) in Raven Pro. We measured three temporal and three spectral parameters (duration, time to the peak of the dominant frequency, time to peak amplitude, highest and lowest frequency, dominant frequency), and both aggregate and average entropy [62], resulting in 8 parameters for the entire vocalization. The four subunits were measured over the bandwidth of the fundamental frequency, to avoid confounding effects of presence or absence of higher harmonics due to recording conditions. Furthermore, giant otter vocalization types vary strongly in emphasizing different harmonics. We choose to measure the fundamental frequency to gain variables comparable among the vocalization types, as well as among the whole vocal repertoire. In each of these four parts, we measured two temporal parameters (time to the peak of the fundamental frequency, time to peak amplitude), three spectral parameters (lowest, highest and peak frequency of the fundamental frequency), one wave form derived parameter (peak amplitude) and the aggregate and average entropy [62], resulting in 8 parameters for each subunit. We converted the values of peak amplitude within the subunits to percentage in relation to peak amplitude of the entire call.

To describe the frequency contour of the vocalizations, we measured frequency and time at 9 points of equal time distance along the fundamental frequency. The 9 points represented start, mid and end point of each subunit. Since the end points of the subunits one, two and three are also the start points of the subunits two, three and four, those points were only measured once. Time point 9 was redundant with the duration of the entire vocalization and was therefore not included. The onset of each vocalization in time was set to zero seconds and was also not included. Thereby, we measured 9 frequency and 7 time parameters to describe the frequency contour. Additionally, we quantified the occurrence of nonlinear phenomena (NLP). Therefore, we measured the proportion of chaos, subharmonics and biphonation in relation to the duration of the entire call. Overall, we obtained 59 acoustic variables to describe each vocalization.

### Statistics

To reduce the number of measured parameters, we conducted a principal component analyses with varimax-rotation. The principal component analyses fulfilled Kaiser-Meyer-Olkin (KMO) and Bartlett’s test criteria and 10 principal components (PCs) with eigenvalues >1 were extracted. We used the 10 PCs to run a stepwise discriminant function analysis (DFA) with leave-one-out cross-validation. Nine of the 10 PCs were included by the DFA. We conducted a one-tailed binomial test to determine whether the DFA classification result differed significantly from a random classification (according to [63]). All statistical tests were performed with SPSS (version 21, SPSS Inc., Chicago, IL, U.S.A.).

## Results

**Figure 1:**
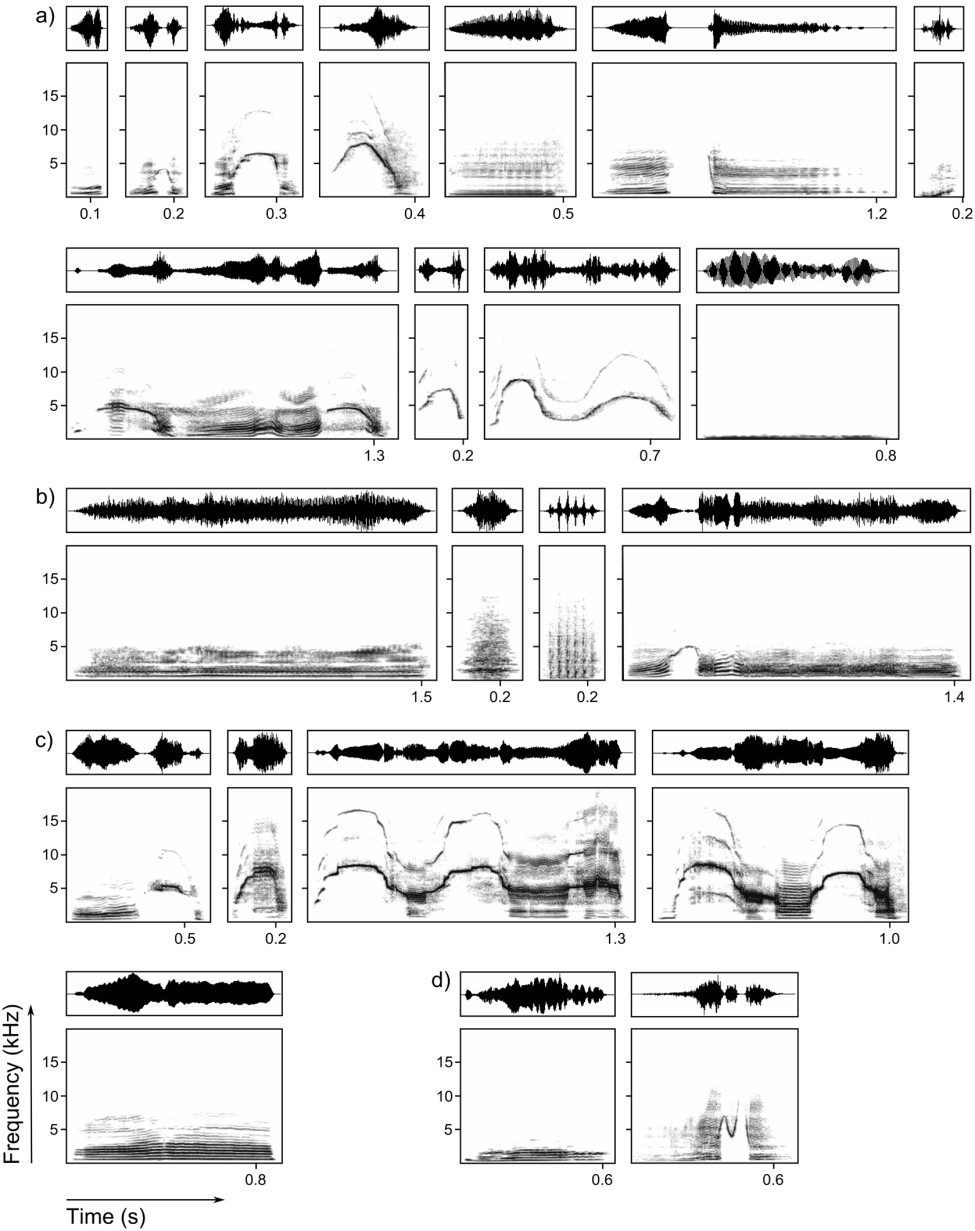
Exemplary calls for the vocal repertoire of giant otters. Calls were obtained from wild and captive individuals. The spectrograms depict frequency over time and were generated using a 1024-point FFT and a Hann window with 75% overlap. The oscillograms show pressure changes over time. a) Cohesion: contact and coordination calls. From left to right: bark, close call, contact call, contact call gradation, hum, hum gradation, hum short, isolation call, whistle, whistle double, and underwater call. b) Alarm calls. From left to right: growl, hah!, snort, and wavering scream. c) Begging calls. From left to right: ascending scream, begging call, begging scream, begging scream gradation, and whine. d) Other calls. From left to right: mating call and suckling call.

**Figure 2:**
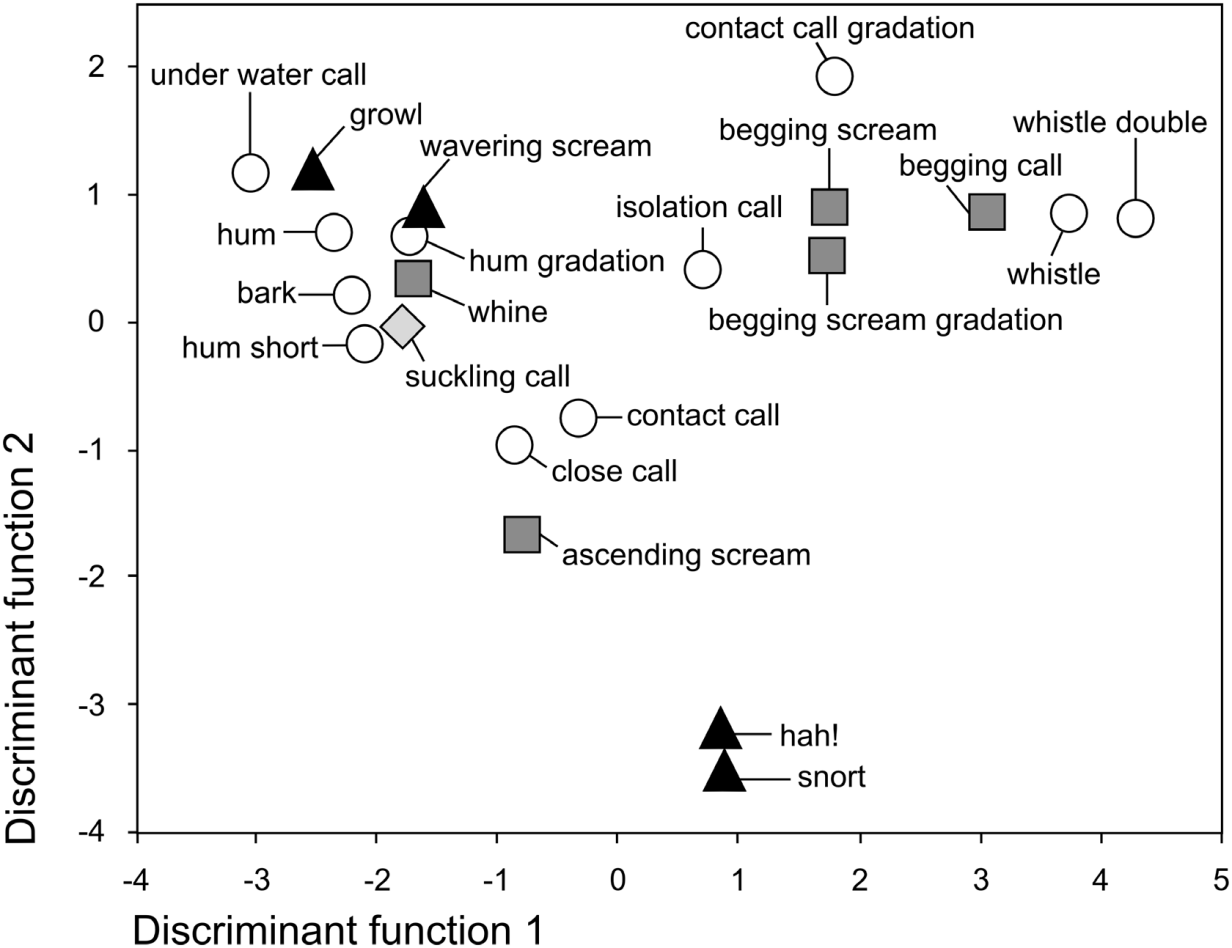
Signal space of the giant otters’ adult vocal repertoire defined by the first two discriminant functions. Symbols indicate group centroids. Discriminant function 1 was mainly shaped by the peak frequency within subunit 1 and the fundamental frequency at 1/3 duration of the entire call (combined in one principal component). Discriminant function 2 was mainly shaped by the average entropy in the subunits 2 and 3 (combined in another principal component).

The vocal repertoire of adult giant otters comprised 22 distinct vocalization types (table 1, figure 1, table S1, table S3, audio S1). Due to its rare occurrence, we did not include the mating call in our statistical analyses. The DFA result supported our classification by assigning the 441 calls to the correct vocalization type in 55.3% of the cross-validated cases (for detailed DFA results, see table 2, figure 2). This classification result differed significantly from a random classification (4.8%; one-tailed binomial test: *p* = 0.0001). The vocalizations were best divided by the peak frequency within subunit 1, the fundamental frequency at 1/3 duration of the entire call (combined in one PC, mainly shaping discriminant function 1), and by the average entropy in the subunits 2 and 3 (combined in another PC, mainly shaping discriminant function 2). Misclassified calls in the cross-validated DFA were mainly misclassified to structurally very similar calls (table 3). For instance, the isolation call was misclassified as belonging to the begging screams in 37.6%. Even though these vocalizations were very similar in structure, only an individual that lost sight of the group emitted isolation calls. Then the group approached the isolated animal, often answering with wavering screams and contact calls. Begging screams were only emitted during intense begging.

**Table 1:** Description of the giant otters’ vocal repertoire and behavioural context.

Cohesion: contact and coordination calls								
Vocalization	Behaviouralcontext	Range	Frequency ofoccurrence	Age		Wild orcaptive		Descriptions byother authors
**Bark**	Calling or greeting group members, swimming or fishing together, moving together on land, playing	Short – moderate	Occasional	Subadults,Adults		Wild and captive		Coo [54]
**Close call**	Calling group members, moving together on land, playing	Short – moderate	Occasional	Juveniles, Subadults,Adults		Captive		Coo call [54]
**Contact call**	Calling group members, swimming or fishing together, moving together on land, playing, begging	Moderate	Very common	Cubs, Juveniles,Subadults, Adults		Wild and captive		Adult call [54], Contact call [40], Grito seco [52], Grito de filhote [52]
**Contact call gradation**	Calling group members, moving together on land, playing, begging	Moderate	Very common	Cubs, Juveniles, Subadults, Adults		Wild and captive		No description
**Hum**	Calling group members, swimming or fishing together, moving together on land, change of direction, resting, playing, scent-marking, soothing	Short	Very common	Cubs, Juveniles, Subadults, Adults		Wild and captive		Hum [40], [42], Hum and Purr [54], Kontaktsummen [43], Murmúrio [52]
**Hum gradation**	Calling or greeting group members, swimming together, moving together on land, change of direction or departure, playing	Short – moderate	Very common	Juveniles, Subadults,Adults		Wild and captive		Coo [42], [52], Coo-hum [54], Let's go hum [96]
**Hum short**	Emerging	Short	Occasional	Juveniles, Subadults,Adults		Wild		No description
**Isolation call**	Calling group members	Large	Occasional	Juveniles, Subadults,Adults		Wild		Cub lost whine [42], Verlorenenruf [43]
**Whistle**	Calling group members, begging	Short	Occasional	Cubs, Juveniles,Subadults,Adults		Wild and captive		Whistle [42]
**Whistle double**	Monitoring the surrounding, begging	Moderate	Rare	Cubs, Juveniles,Subadults		Wild and captive		No description
**Underwater call**	Diving	Unknown	Unknown	Unknown		Wild and captive		Unterwasserlaute [53]
**Alarm and threatening vocalizations**								
**Vocalization**	**Behavioural** **context**	**Range**	**Frequency of** **occurrence**	**Age**	**Wild or** **captive**		**Descriptions by** **other authors**	
**Growl**	Defending a fish, threatening or warning, playing	Moderate	Very common	Cubs, Juveniles,Subadults, Adults	Wild and captive		Adult growl [54], Growl [42], Knurren [43]	
**Hah!**	Warning, submission	Short	Occasional	Cubs, Juveniles,Subadults, Adults	Wild and captive		Hah [42], [54]	
**Snort**	Alarm, threatening or warning	Moderate - large	Very common	Cubs, Juveniles,Subadults, Adults	Wild and captive		Snort [42], [52], [54], Warnschnauben [43]	
**Wavering scream**	Alarm, begging	Large	Occasional	Subadults, Adults	Wild and captive		Adult scream [54], Grito ondulado [52], Schreien [43], Wavering scream [42]	
**Begging calls**								
**Vocalization**	**Behavioural** **context**	**Range**	**Frequency of** **occurrence**	**Age**	**Wild or** **captive**		**Descriptions by** **other authors**	
**Ascending scream**	Begging, stealing prey	Moderate - large	Common	Juveniles, Subadults, Adults	Wild and captive		No description	
**Begging call**	Begging	Moderate	Very common	Cubs,Juveniles, Subadults,(Adults)	Wild and captive		Bettelgeschrei [43], Cub call [54], Food begging call [42], Grito de filhote [52]	
**Begging scream**	Begging	Large	Very common	Juveniles,Subadults, (Adults)	Wild and captive		Bettelgeschrei [43], Cub begging scream [54], Food begging call [42], Grito de filhote [52], Squeak [54]	
**Begging scream gradation**	Begging	Large	Common	Cubs, Juveniles, Subadults, (Adults)	Wild and captive		Adult and cub high scream [54], Bettelgeschrei [43], Food begging call [42]	
**Whine**	Begging, Alarm	Moderate - large	Common	Juveniles, Subadults, Adults	Wild and captive		Grito ondulado [52]	
**Other**								
**Vocalization**	**Behavioural context**	**Range**	**Frequency of** **occurrence**	**Age**	**Wild or** **captive**		**Descriptions by** **other authors**	
**Mating call**	Copulation	Moderate	Rare	Adults	Wild and captive		No description	
**Suckling call**	Nursing	Moderate	Occasional	Cubs, Juveniles,Subadults	Wild and captive		Nursing hum [59], Scream-gurgle [54]	

**Table 2:** Assessment of model fit of the discriminant function analysis.

Function	Eigenvalue	% of Variance	Test of Function	Wilks' Lambda	Chi-square	df	P
1	4.314	38.2	1 to 9	0.002	2596.028	180	<0.0001
2	2.205	19.5	2 to 9	0.012	1886.126	152	<0.0001
3	1.639	14.5	3 to 9	0.038	1391.124	126	<0.0001
4	1.176	10.4	4 to 9	0.100	978.774	102	<0.0001
5	0.990	8.8	5 to 9	0.218	648.253	80	<0.0001
6	0.447	4.0	6 to 9	0.433	355.809	60	<0.0001
7	0.227	2.0	7 to 9	0.626	198.741	42	<0.0001
8	0.181	1.6	8 to 9	0.769	111.866	26	<0.0001
9	0.102	0.9	9	0.908	41.154	12	<0.0001

**Table 3:** Percentage of misclassified cases in the cross-validated DFA, listing the vocalizations with which the misclassified cases have been confused by the DFA*.

Call (n = number of cases)	Cross-validated cases	
	Correctly classified (%)	mainly misclassified to (n = number of misclassified cases)*
Bark (n = 21)	38.1	Hum (n = 8)
Close call (n = 10)	30.1	Ascending scream (n = 2), contact call (n = 2)
Contact call (n = 32)	62.5	**
Contact call gradation (n = 22)	88.0	**
Hum (n = 30)	70.0	**
Hum gradation (n = 29)	65.5	**
Hum short (n = 9)	33.3	Bark (n = 6)
Isolation call (n = 16)	43.8	Begging scream (n = 3), begging scream gradation (n = 3)
Whistle (n = 18)	77.8	**
Whistle double (n = 13)	53.8	**
Underwater call (n = 9)	0.0	Hum (n = 5)
Growl (n = 29)	55.2	**
Hah! (n = 17)	52.9	**
Snort (n = 32)	71.9	**
Wavering scream (n = 11)	18.2	Growl (n = 4)
Begging call (n = 21)	76.2	**
Begging scream (n = 32)	50.0	Begging scream gradation (n = 7)
Begging scream gradation (n = 30)	33.3	Begging call (n = 5)
Ascending scream (n = 25)	60.0	**
Whine (n = 20)	60.0	**
Suckling call (n = 12)	0.0	Growl (n = 4)

* given for calls correctly classified less than 50.0%. ** Original vocalization was correctly classified in >50.0% of the cases.

**Figure 3:**
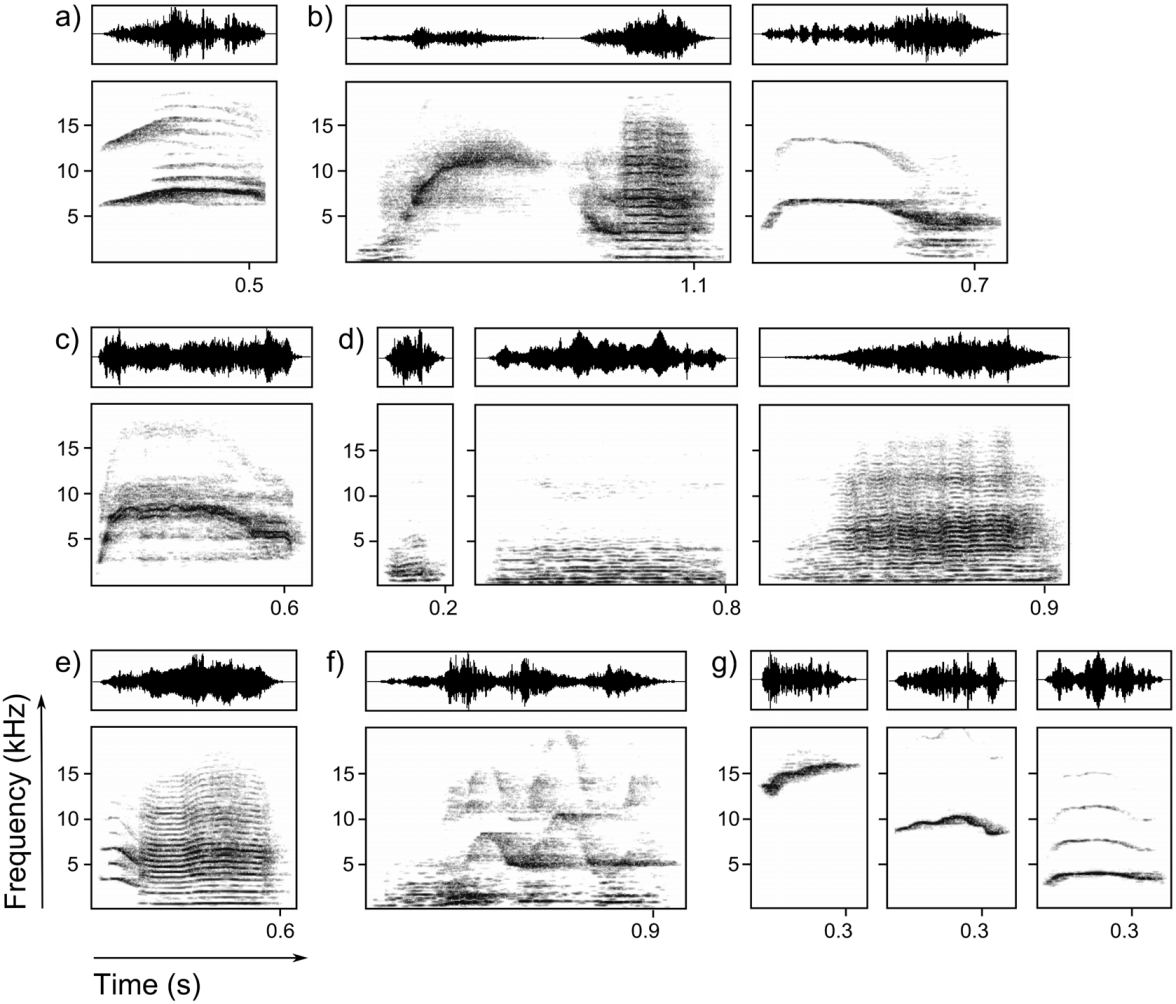
Exemplary calls from neonate giant otters. Vocalizations were recorded from one wild and one captive litter. The spectrograms depict frequency over time and were generated using a 1024-point FFT and a Hann window with 75% overlap. The oscillograms show pressure changes over time. a) Begging call-like vocalization. b) Contact call-like vocalizations. c) Distress call 1. d) Hums. From left to right: bark-like call, hum-like call, and distress call 2. e) Hum gradation-like vocalization. f) Suckling call. g) Whistles. From left to right: high whistle, whistle, and low whistle.

We found 11 structurally distinguishable vocalizations within the vocal repertoire of new born cubs (figure 3, table 4, audio S2). The neonate vocalizations included begging call-like and contact call-like vocalizations, distress calls, hum-like vocalizations, whistles, and the suckling call described below. We found no equivalent to the high whistle and the distress calls in the adult repertoire.

**Table 4:** Structural description of the neonate giant otter vocal repertoire.

Vocalization	Description	Category	Frequency ofoccurrence	Wild orcaptive	Descriptions byother authors
**Begging call-like** **vocalization**	Resembles the adultbegging call.	‘BC-like’	Common	Captive	No description
**Contact call-like** **vocalization**	Resembles the adultcontact calls.	‘CC-like’	Occasional	Wild and captive	No description
**Distress call (1)**	No equivalent in adultvocalizations. Emittedwhen cubs are movedfrom one den to another.	‘DC-like’	Rare	Wild and captive	Newborn cub squeaks [42], Jungenfiepsen [43]
**Hum gradation-** **like vocalization**	Resembles the adult humgradation. Combinationof a hum and a contact call.	‘Hum gradation’	Common	Captive	No description
**Bark-like call**	Resembles theadult bark vocalization.	‘Hums’	Common	Captive	No description
**Distress call (2)**	No equivalent in adultvocalizations. Hum withaccentuation of higherharmonics.	‘Hums’	Common	Captive	Cub scream [54]
**Hum-like vocalization**	Resembles theadult hum.	‘Hums’	Very common	Captive	No description
**Suckling call**	Suckling. Onlyemitted when nursed.	‘Suckling call’	Occasional	Wild and captive	Scream gurgle [54], nursing hum [59]
**High whistle**	No equivalent in adultvocalizations. Pure tonewith F_0_ above 10 kHz.	‘Whistles’	Common	Captive	No description
**Whistle**	Resembles the adultwhistle. Pure tone withF_0_ between 5 kHz and10 kHz.	‘Whistles’	Common	Captive	No description
**Low whistle**	Resembles the adultwhistle. Pure tone with F_0_ below 5 kHz.	‘Whistles’	Common	Captive	No description

### Structure and behavioural context of the vocalizations

In table 1, we give a brief overview of the distinct vocalizations within the vocal repertoire. We described the calls found in our study in four major behavioural categories: a) cohesion, including all vocalizations emitted in the context of staying in contact and coordination of the group, b) alarm and threatening vocalizations, referring to warning or threatening behaviour, c) begging vocalizations, and d) other contexts such as mating and nursing. Within these categories, we listed the calls alphabetically. The examination of the behavioural context revealed that giant otters produce structurally similar vocalizations in various situations, as well as using different vocalizations in an apparently similar context.

### Cohesion calls

In the context of group cohesion, we found 11 distinct vocalizations, which we defined as bark, close call, contact call, contact call gradation, hum, hum gradation, hum short, isolation call, underwater call, whistle and whistle double. Several of these vocalizations seemed to be structurally derived from the contact call.

*Bark*. This is a very short, constant frequency vocalization, resembling the constant frequency parts of the contact call. We recorded it in contexts of movement (swimming or fishing together, moving together on land), group cohesion (calling or greeting group members) and playing, but could not detect its detailed function. *Close call*. This call is structurally very similar to the contact call, but shorter and the maximum frequency is lower compared to the contact call. It is produced as a cohesion call in close contact (moving together on land, calling group members, playing). *Contact call*. This modulated call starts and ends with a quasi-constant frequency part and has a whistle-like part in the middle. Its main function is to stay in contact with group members and to coordinate group movements. It is produced in situations of visual separation, for instance during fishing by one individual above the surface, while the others are still diving, or when group members are separated over larger spatial distances, as well as in any other context of movement. It may also be included in begging bouts or emitted during play. *Contact call gradation*. This call is structurally very similar to the contact call, but lacks the first quasi-constant frequency part and thus, starts with the whistle-like part. Like the contact call and the close call, it is produced in the context of cohesion (moving together on land, calling group members, playing). It is more often emitted within begging bouts than the contact call. *Hum*. This constant frequency vocalization has a variable duration. It is a short-range contact and cohesion call of low volume, frequently emitted in group activities (swimming or fishing together, moving together on land, resting, playing, scent-marking). It is also used to soothe group members in social interactions or when a group passes the boat of the observer. *Hum gradation*. The structure of the hum gradation reaches from a strongly accentuated hum to a call combination of a hum and a contact call. The contact call part of this vocalization can be included at the beginning, middle or end of the hum. The hum gradation, like hum and contact call alone, was produced in the context of group cohesion. We mainly recorded it when the group changed its direction, or when group members met. The functions of hum (cohesion in close contact and soothing) and contact call (contact, cohesion over larger distances, or reunion after separation) seem thereby combined to the slightly different and more specific meaning of directional change. In terms of group reunion, this call combination can signal individuality and friendly intent by soothing the approaching individuals. *Hum short*. This call is structurally very similar to the bark, but only heard when a giant otter emerges after diving. Other authors have not yet described this call, and we recorded it only from wild giant otters. *Isolation call*. This loud piercing scream is similar in structure to the begging scream gradation and the wavering scream. It is only emitted by individuals who lost sight of the group. The group may answer with wavering screams and contact calls, changing to hums and hum gradations when reaching visual distance. Especially inexperienced juvenile giant otters often get separated from the group during fishing. As long as their fishing and swimming abilities are not fully developed, they tend to eat prey on fallen trees at the lakeshore, whereas the group might move on fishing (own observation). We listed the isolation call in the behavioural context of group coordination and cohesion, since its emission by an isolated individual will result in answering and approaching behaviour of the group. Nevertheless, the call structure applies to distress calls, belonging to assembly alarm vocalizations [12]. The tonal and modulated isolation call is repeated until the group responds and thus, it is designed to attract the attention of group members, likely encoding the location of the lost individual and the urgency of the situation. *Whistle*. This short, modulated and tonal call is less audible than the contact call. Since we recorded it mainly in situations of movement, especially with adults whistling and their cubs following them subsequently, we assume it to be a contact call as well, even though we could not detect its detailed function. *Whistle double*. This rarely emitted vocalization is tonal with two modulations. It was mainly produced while monitoring the surroundings and sometimes emitted during begging. We could not detect its detailed function. *Underwater call*. In the wild, as well as in the zoos, we only recorded one distinct underwater vocalization. This very low frequency call resembles the air-borne hum and we assume it to function as a cohesion call as well, probably to ease coordination of movements during hunting underwater.

### Alarm and threatening vocalizations

Alarm and threatening vocalizations were the growl, hah!, snort, and the wavering scream. Especially screams and alarm signals included the ‘sound of arousal’ [64], i.e. nonlinear phenomena.

*Growl*. This typical carnivore threat growl has a quasi-constant frequency and is graded according to the intenseness of arousal. Its main function is to defend a fish against begging group members, but it is also emitted in a playing context. In a submissive context, offspring greeting their parents may emit a soft growl after a hah!. *Hah!*. The hah! is a short and sharp exhalation. Giant otters emit the hah! when detecting unusual objects, animals or humans. It might function as a warning of group members against intruders or humans. In a submissive context, offspring greet their parents shaking the head with open mouth and emit the hah!, sometimes followed by a soft growl. *Snort*. The snort is an elongated and accentuated sharp exhalation with a varying number of pulses. It is an alert alarm signal to warn group members against caimans, humans, or any other potential danger, but is also used to threaten conspecifics in conflicts over food or dominance rank. As an alarm call, intermediate between assembly and alert signals, monitoring or periscoping otters emitted these calls to inform group members about potential danger. *Wavering scream*. This call is a loud, sometimes more howl-like, sometimes more piercing modulated scream. It mainly serves as an assembly call, uttered during caiman interactions or acoustic territorial marking and can be heard over a large distance. It is also emitted as an answer to isolation calls. Often group members scream simultaneously with one individual emitting the first scream and the rest of the group joining into a chorus. Single or repeated wavering screams also occur in begging bouts.

### Begging vocalizations

The vocalizations emitted in the context of begging behaviour included the ascending scream, the begging call, begging scream and begging scream gradation, and the whine.

According to our observations, begging vocalizations uttered in begging bouts always elicit a response from the individual with a fish. The response might be giving the fish to the begging individual or defending the fish and refusing to provide it. The denial is often accompanied by growls. In case of a denial to share, the begging individual might intensify its begging vocalizations or might even try to steal the fish. All begging vocalizations occurred in the penetrative begging bouts of juvenile giant otters. A bout usually started with single begging calls and was intensified with screams and interspersed with contact call gradations when the cub detected an adult with fish. When receiving or stealing prey from a group member, the calls changed from the begging calls and screams to the ascending scream and the whine, often accompanied by defence displays such as kicking with the hind legs, tail waving and growling at all others in close vicinity. Even adults produced begging vocalizations. In captive groups, all individuals engaged in a piercing begging chorus at feeding time. In the wild group at Cocha Cashu, we recorded begging of the aging mother directed at her youngest son (see video S1), a behaviour previously described by Davenport [65].

*Ascending scream*. The structure of the ascending scream resembles a contact call with an elongated and ascending first part. This call is emitted within begging bouts or when stealing a fish from a group member, the latter accompanied by defensive body postures like kicking with the hind feet and tail wagging after a successful fish over-take. *Begging call*. This short modulated calls has an elaborate number of subharmonics. Begging bouts often start with one or several begging calls, followed by the other begging vocalizations in alternating or variable order. *Begging scream*. This call is a piercing, modulated scream with subharmonics and often chaotic parts, with a very variable structure. Begging individuals use this scream to intensify their begging behaviour. *Begging scream gradation*. This piercing, modulated screams often has subharmonics or chaotic parts. The structure and frequency modulation is very similar to that of isolation calls. Begging individuals use this scream to intensify their begging behaviour. *Whine*. This howl-like, quasi-constant frequency call has a variable duration and resembles the wavering scream. Like the ascending scream it is produced within begging bouts or when stealing a fish from a group member, the latter accompanied by defensive body postures. In wild groups, the whine was also produced as an alarm call during caiman interactions.

### Other contexts

Mating and nursing were the two other behavioural contexts in which we recorded giant otter vocalizations. *Mating call*. This growl-like vocalization has a higher fundamental and more modulated frequency than other growls. We recorded mating calls only from one alpha female, copulating in the water while the male emitted growls. *Suckling call*. This blatant vocalization resembles a hum with additional tonal parts. Generally, only cubs and juveniles emit the suckling call while nursing, but in the Tierpark Hagenbeck group, we recorded suckling calls of subadults nursing after the death of a new born litter.

## Discussion

When comparing our findings from Peruvian and captive giant otters to the vocal repertoire of giant otters from Brazil [54], the uniformity of the vocalizations and the related behavioural context is striking. Our classification only slightly differs from Leuchtenberger [54]. Therefore, we think that the vocal repertoire is generally applicable to all local populations. Vocal differences will rather be found within distinct vocalization types among groups, families, sexes or individuals.

Even though we only measured source-induced acoustic parameters, the DFA result supported our classification of the adult vocal repertoire. We interpret the misclassification of structurally very similar vocalizations being a result of a close structural resemblance, conveying different meaning depending on the situation in which the vocalizations are produced (sensu [21]). We could relate signal structure and function in giant otter vocalizations to the motivational-structural rules described by Morton [11] and Bradbury and Vehrencamp [12]. The vocalizations of giant otters resemble the overall structure of canid [66] and other mammalian [21] vocal repertoires. The giant otter repertoire presents a continuum of distinct and graded vocalizations with variants, encoding information on internal state or external events. While the number of vocalizations a species can produce is limited by anatomical constraints [1], [4], vocal complexity can be enhanced by call combinations [67] or segmental concatenation, as has been found in banded mongooses, *Mungos mungo* [17]. Like otters, Herpestids show a wide spectrum of social organization from solitary to highly social group living species [35]. Banded mongooses and meerkats, *Suricatta suricatta*, are obligatory social species, showing a strong group cohesion, cooperative foraging and breeding [68]. Due to the similar structure and function of the giant otters contact call to close calls of banded mongooses [17], we propose that the contact call and structurally similar vocalizations may also include segmental concatenation, which is the combination of different vocal cues in one call type [17]. The individual distinctiveness of giant otters contact calls is mainly encoded in the second constant frequency part and in the frequency contour of the modulated part [40]. In banded mongoose close calls, the discrete individual cues are encoded in the first call segment, whereas the behavioural context is embedded as a graded cue within the tonal part of the call [17]. Since we [40] did not test for other cues than vocal individuality, it may well be that giant otters combine individual signatures and graded behavioural cues in the contact call.

With the results of our underwater recordings, we cannot support the findings of several underwater vocalizations by an earlier study [53]. Air-borne calls can be recorded underwater, since the giant otters’ thorax and throat are submerged while swimming. This may lead to misinterpretation of air-borne calls as being produced while diving.

Among snorts, giant otters seem to be able to differentiate the type of signalled danger depending on the situation [43]. The number of pulses and production rate of snorts could refer to the internal state of arousal or the estimation of the external threat perceived by the signaller, as well as signalling the exact type of danger (such as caimans, humans, or non-group members). According to the signal design rules by Bradbury & Vehrencamp [12], the localization of the sender of alert signals can be enhanced by repeated, short and pulsed broadband vocalizations. By varying the repetition rate, senders can inform group members about the urgency of the situation [12]. Reference and emotion can be combined in one call [69]. The content of information and its interpretation depend on the signaller’s or the receiver’s point of view [69]. By adding unexpected and unpredictable components to alarm calls, nonlinear phenomena could reinforce attention in the receivers, and therefore decrease habituation [64]. The same applies to the wavering scream, but its function needs to be analysed in more detail. It might just signal high arousal, or, as a referential vocalization provide exact information about the external threat perceived. Generally, assembly alarm calls provide information on the spatial location of the group and may encode group specific cues [12]. Several times, we observed giant otter groups screaming in a chorus while patrolling the lakeshore, but we could not detect caimans or any other obvious threat. Therefore, we think that giant otter chorus calling may not always signal caiman presence, but could also represent acoustic territorial signalling. Other group-territorial species like lions or wolves engage in chorus calling, providing information on group size, their presence as territory holders and thereby their resource holding potential to neighbours or potential intruders [12], [70]–[72].

During development, giant otter cubs need to learn how to capture prey on their own. As long as they are depended on food sharing from elder group members, they engage in intense begging for food [43]. A comparable begging system with dependant offspring emitting diverse begging vocalizations can be found in the cooperatively breeding meerkat [73], [74]. Meerkat cubs follow their group while foraging and their vocalizations change when observing an adult with food. The intenseness of the vocalizations serves to attract the feeders attention and to ‘outcall’ siblings [74]. Since this intense calling is energetically costly, the authors hypothesize that the cubs only increase begging when the chance to receive food is optimal [74]. Like meerkat pups, giant otter cubs are mobile and benefit from food provision by adults, from signalling their spatial location and from ‘outcalling’ their siblings. Furthermore, giant otter cubs may benefit from adding nonlinear phenomena to their begging calls, keeping potential feeders from habituating to the piercing screams [64], [75]. Nonlinearities may also provide honest information about the internal state of arousal [64], [75], thus denoting condition [76].

Neonate giant otters are vocally active from birth on. Structure and usage of giant otter vocalizations seem to undergo comparable changes as for instance in wolves and wild dogs [16], [77]. The distress calls and the high whistle do not persist until adulthood. The other vocalizations we found in new born cubs are likely to be precursors of the distinct adult vocalizations. Giant otter cubs emit the full vocal repertoire not later than at an age of 3 months, whereas the lowering of the fundamental frequency continues until six to 12 months (own observation). The babbling bouts of neonate giant otter cubs very likely fulfil the two main purposes of vocal practise and social care solicitation [25], [26], [28]. The general structure of the vocalizations in neonate babbling bouts was comparable to adult vocalizations, but showed some subtle differences. For instance, the duration of cub contact call-like vocalizations was extended, and the calls often included gaps. We suggest that the cubs need to practice the shift from the constant frequency part to the modulated frequency and back. Concerning the social organization of giant otters, we expect that babbling also serves to intensify social interactions and caregiving in giant otters. This function of babbling was reported for the social and cooperatively breeding pygmy marmoset, *Cebuella pygmaea* [25], [26], [28]. Giant otter cubs may switch from babbling to begging vocalizations and isolation calls as care eliciting cues during development.

When comparing social organization among the otter species, we come to the same conclusion as Manser et al. [68] in their comparative study on vocal complexity in Herpestids. Group size alone, as a measure for social complexity, cannot explain the variation of vocal repertoire size in otters. Still, knowledge on the acoustic communication of the 13 otter species is scarce, so that we restrict the comparison to eight out of 13 otter species (table S2, see also [78] on species comparisons). Vocalizations are reported for eight species and for only five of them exist published descriptions of the vocal repertoire [42], [55], [79]–[85].

The solitary and solitary foraging Neotropical otter, *Lontra longicaudis* [41], [83] has more than four vocalization types [83]. The Eurasian otter, *Lutra lutra*, produces 8 basic vocalization types with some variants [79], [80]. Quaglietta et al. [86] recently gave new insight into the social organization of Eurasian otters, revealing a more social lifestyle than previously reported, so that we list it here as semi-solitary. Social interactions among Eurasian otters are enhanced by the overlap of territories, formation of dyads and sharing of resting sites. Quaglietta et al. [86] discuss their findings in relation to environmental factors and otter density. The semi-solitary Cape clawless otter, *Aonyx capensis*, has approximately 13 [55] and the gregarious sea otter, *Enhydra lutris*, 10 vocalization types [81]. North American river otters, *Lontra canadensis*, showing a variable social structure and cooperative foraging [41], [87], emit 11 distinct vocalization types (four distinct vocalizations with seven subtypes) [85]. Male groups of North American river otters may include up to 30, and male groups of sea otters may include even up to 2000 animals [41], [88], [89]. Although the group size of both species exceeds the number of individuals reported for giant otter groups [42], the repertoire of North American river otters and sea otters shows less distinct vocalizations [81], [85] than we found in giant otters. Therefore, other factors influencing the social organization of a species need to be taken into account. The large aggregations of male sea otters (‘rafts’) occur during the nonbreeding season, whereas female groups are much smaller and family units consist of mother and cub. Territorial males may attend mother-cub pairs, but do not help in rearing the offspring [88]. This behavioural pattern is due to the marine habitat of sea otters, as well as due to a conflict between territoriality and gregariousness, resulting in an otter-unusual social organization [41]. In North American river otters, variability of social organization is also based on regional habitat differences [41]. Depending on the region, territorial overlap occurs among females, among males, or among the two sexes. River otters may group together in ‘packs’ of related or unrelated individuals for several months [41].

The more social species of smooth-coated otter, *Lutrogale perspicillata* [90], has at least four distinct vocalization types, but the vocal repertoire description is only anecdotal [56]. Asian small-clawed otters, *Aonyx cinerea*, showing a social organization very similar to giant otters [41], [91]–[93], were described to have about 12 vocalization types by Timmis [82]. Lemasson et al. [84] classified four main context-dependent vocalization units (7 distinct vocalization types with gradations) for Asian small-clawed otters, resembling the four major behavioural categories we found in giant otters.

Group formation and composition in giant otters are influenced by several factors originating from life history and habitat effects [45], [94] and may lead to high variation of relatedness within giant otter groups [45]. Changes in structure of established groups mainly result from the disappearance of one partner of the reproductive pair. Dominant females are thereby replaced by a philopatric female group member, whereas males tend to migrate into another existing group [45], [94]. Offspring of the former couple are adopted by the new group member [45], [94]. Group size and reproductive success seem to be directly linked to lake size of a territory [94]. If all high quality territories in an area are already inhabited by resident groups, dispersing individuals may form temporary groups in less suitable habitats [45], [94].

Besides the plasticity in group composition in giant otters, the basic family group of a breeding pair with offspring of several years likely promotes long-term individual relationships and diversity of social roles in giant and Asian small-clawed otters. These aspects complement to the complexity of a social system [29]. Vocal complexity in giant and Asian small-clawed otters is enhanced by graded vocalizations, and probably by segmental concatenation and call combination. Cohesion calls are individually distinct and both species show vocal individual discrimination [40], [84], [95], thus, adding an individual scale to their vocal and social complexity.

A more sophisticated vocal repertoire with increasing group size can therefore not fully be supported in otters. On the other hand, the social organization and the strength of social relationships within otter groups may account for their vocal complexity, reflecting an underlying pattern of coevolution of social and vocal complexity in otters, as has been shown for primates [30], [31], canids [36], [66] and herpestids [35]. Nevertheless, new insight into the social organization and communication of otters may still change our classification (compare [68], [78], [86]). Consistent with the recent knowledge on otter sociality and acoustic communication, we like to emphasize that giant otters still belong to the socially most complex species and that their social complexity is reflected in their sophisticated vocal system. We hope that this detailed account of the giant otters’ vocal repertoire will help to facilitate conservation efforts of this endangered species. By providing the behavioural context and acoustic measures of the vocalizations, the otters’ arousal or stress in a given situation may be easier to evaluate.

In conclusion, we found a sophisticated vocal repertoire of adult giant otters, with 22 distinct vocalizations. Thus, the giant otters’ social complexity seems to be reflected in their vocal complexity. Neonate vocalizations were also complex and included precursors of the adult repertoire, as well as age-specific calls, supporting our hypotheses on the vocal repertoire of neonate giant otters. Moreover, the relationship of signal structure and function in giant otter vocalizations generally follows the expectations stemming from Morton’s motivational-structural rules.

## Supporting Information

Table S1 **Mean values (±SD) for the most important variables characterizing distinct vocalizations within the giant otters’ vocal repertoire.**
(DOCX)

Table S2 **Comparison of social organization and vocal repertoire size in eight of the 13 otter species.**
(DOCX)

Table S3 **List of all 441 adult giant otter vocalizations measured in this study, including origin of the individuals, vocalization type, behavioural context and vocal parameters.**
(XLSX)

Video S1 **Begging, contact, group reunion and contact over larger distance (Lake Cocococha, Peru, 2012).** Juveniles are swimming close to the lakeshore and beg, while the family is swimming in the centre of the lake. In sec 5, the family approaches the begging juveniles (sec 38), the adults provide them with fish (sec 44) and emit contact calls (contact calls sec 18.5, 53.5 and 58.0). At min 1 the family is fishing again in the centre of the lake, but still in vocal contact with the juveniles.
(MP4)

Video S2 **Group reunion (Lake Cashu, Peru, 2011).** The juvenile is swimming alone behind the riparian vegetation, while the family is resting on a trunk (sec 5). At sec 13.5, the family starts to call for the juvenile and approaches him (contact calls sec 13.5, 16.5, 17.5, hum gradation sec 18.5 and 21.5, hums sec 25 and 27).
(MP4)

Video S3 **Contact, group cohesion and coordination (Lake Sandoval, Peru, 2012).** The giant otter family is swimming together, they emit hums, contact calls and a hum gradation, initializing a change of direction and to go on fishing.
(MP4)

Video S4 **Territorial marking (Lake Salvador, Peru, 2011).** The alpha couple is emitting hums while marking the entrance of the den by cleaning and smoothing the floor.
(MP4)

Video S5 **Soothing hums (Lake Salvador, Peru, 2011).** The giant otter family is exploring the hiding place of the observers’ boat. The otters are intensely observing and sniffing at the hiding place of our boat and examine the trunk, where we installed a flagging, while emitting hums to soothe each other during this unusual encounter.
(MP4)

Video S6 **Wavering screams in an isolation context (Lake Salvador, Peru, 2011).** Two juveniles have been separated from the group. One emits a contact call, the other one an isolation call (sec 2), the group answers with wavering screams and approaches the juveniles. When they are closer, they emit contact calls (sec 20).
(MP4)

Video S7 **Growling to defend a fish (Lake Sandoval, Peru, 2012).** The adult giant otter sitting on the trunk is eating a large fish. He is growling to threaten his group members and to defend his fish against them. At min 1 he emits an accentuated growl against a group member.
(MP4)

Video S8 **Minor alarm (Lake Cocococha, Peru, 2012).** The alpha male leaves the den and emits hah!s when detecting the human observer (hah! Sec 1.5 and 6).
(MP4)

Video S9 **Snorting at each other, hah! in a submissive context (Lake Salvador, Peru, 2011).** The eldest daughter of the family is marking branches by scratching the leaves. In second 9, the mother comes and threatens her by body posture, the daughter shows the submissive gesture, exposing her throat and hah!ing with open mouth (sec 10). Then the mother starts to scratch the leaves (sec 14). Sec 33: again, the daughter is marking leaves but she turns away as she sees the mother coming (sec 38). The mother snorts at her (sec 39), the daughter swims away and the mother marks the leaves.
(MP4)

Video S10 **Snorting at a caiman (Lake Salvador, Peru, 2011).** The group is fighting against juvenile caimans which rest at the lakeshore.
(MP4)

Video S11 **Caiman alarm (Lake Cashu, Peru, 2011).** One otter detected the very huge caiman, living in this area of the lake, the group members answer his alarming wavering screams with contact calls.
(MP4)

Video S12 **Group screaming in alarm (Lake Sandoval, Peru, 2012).** The context could not be examined, it could be the close presence of the tourist boat, as well as a caiman detected underwater.
(MP4)

Video S13 **Characteristic begging (Lake Tres Chimbadas, Peru, 2012).** One giant otter is sitting on a trunk and eating a fish. A juvenile comes close and emits begging calls, interspersed with screams (wavering scream sec 7 and 32, begging scream gradation sec 40 and 42).
(MP4)

Video S14 **Begging behaviour of the ageing alpha female directed at her youngest son (Lake Cashu, Peru, 2011).** The juvenile is sitting on a trunk and eating a fish. At sec 12, the mother approaches him, climbs the trunk (sec 19) and begs for the fish (begging calls and a begging scream gradation from sec 20). At sec 27 she starts to steal the fish from her offspring. When she emerges with the fish, she emits a growl and an ascending scream (sec 32).
(MP4)

Audio S1 **Giant otter adult vocal repertoire, including audio files AS001 to AS441 as listed in table S3.**
(ZIP)

Audio S2 **Neonate giant otter vocal repertoire, including audio files AS442 to AS453, as listed in** **figure 3** **and table S3.**
(ZIP)
