# Supplementary material for: The Vocal Repertoire of Adult and Neonate Giant Otters (Pteronura brasiliensis)
Source: PLoS One. 2014 Nov 12;9(11):e112562. doi: 10.1371/journal.pone.0112562 (PMC4229255; doi:10.1371/journal.pone.0112562)
Supplement: Table S1 — Mean values (±SD) for the most important variables characterizing distinct vocalizations within the giant otters’ vocal repertoire. (DOCX) [file pone.0112562.s001.docx]

Table S1: Mean values (±SD) for the most important variables characterizing distinct vocalizations within the giant otters’ vocal repertoire.

| **Call (n = number of cases)** | **Parameters mainly shaping discriminant function1** | | **Parameters mainly shaping discriminant function2** | | **Entire call** | | | | |
| --- | --- | --- | --- | --- | --- | --- | --- | --- | --- |
|  | **peak frequency part1 (Hz)** | **fundamental frequency at 1/3 duration of the call (Hz)** | **average entropy part2** | **average entropy part 3** | **duration (s)** | **minimum frequency kHz)** | **maximum frequency (kHz)** | **peak frequency (Hz)** | **time to peak frequency (s)** |
| Ascending scream (n=25) | 395.3 (±73.3) | 386.3 (±47.7) | 2.232 (±1.033) | 2.698 (±0.727) | 0.607 (±0.382) | 196.3 (±42.6) | 15010.6 (±15010.6) | 3603.7 (±2209.9) | 0.394 (± 0.377) |
| Bark (n=21) | 300.7 (±126.6) | 284.0 (±81.6) | 1.401 (±0.263) | 1.461 (±0.274) | 0.198 (±0.093) | 114.5 (±54.9) | 5038.4 (±5038.4) | 688.6 (±477.8) | 0.103 (± 0.056) |
| Begging call (n=21) | 5522.3 (±1914.1) | 7351.9 (±1605.7) | 2.203 (±0.717) | 2.433 (±1.133) | 0.279 (±0.053) | 346.2 (±309.1) | 18086.3 (±18086.3) | 6566.9 (±2657.2) | 0.106 (± 0.074) |
| Begging scream (n=32) | 4772.4 (±2852.5) | 4086.2 (±3599.9) | 2.349 (±1.039) | 2.452 (±1.006) | 0.985 (±0.439) | 308.7 (±200.4) | 19322.7 (±19322.7) | 5226.5 (±2720.2) | 0.449 (± 0.326) |
| Begging scream gradation (n=30) | 4716.8 (±3205.5) | 5662.5 (±3663.1) | 2.592 (±0.887) | 2.329 (±0.897) | 0.665 (±0.218) | 357.3 (±354.4) | 18608.5 (±18608.5) | 5443.7 (±2092.1) | 0.419 (± 0.225) |
| Close call (n=10) | 1183.6 (±1439.8) | 1659.4 (±1876.5) | 2.300 (±1.059) | 2.600 (±0.843) | 0.221 (±0.076) | 173.3 (±60.2) | 12677.6 (±3376.0) | 2245.3 (±1231.6) | 0.129 (± 0.083) |
| Contact call (n=32) | 1334.4 (±1910.5) | 2454.1 (±2894.8) | 2.323 (±0.613) | 2.391 (±0.893) | 0.346 (±0.076) | 173.3 (±53.0) | 13105.6 (±13105.6) | 2854.9 (±2508.6) | 0.189 (± 0.117) |
| Contact call gradation (n=22) | 6296.2 (±2057.8) | 6978.5 (±2113.4) | 2.131 (±0.616) | 2.706 (±0.791) | 0.247 (±0.126) | 268.8 (±220.2) | 14768.8 (±14768.8) | 4231.8 (±2354.1) | 0.146 (± 0.103) |
| Growl (n=29) | 284.9 (±76.6) | 279.6 (±96.6) | 1.318 (±0.136) | 1.304 (±0.145) | 1.557 (±1.024) | 121.9 (±50.8) | 5768.7 (±5768.7) | 952.0 (±794.7) | 0.870 (± 0.933) |
| Hah! (n=17) | 375 (±93.8) | 359.3 (±97.6) | 3.809 (±1.220) | 3.691 (±1.201) | 0.191 (±0.060) | 185.7 (±90.7) | 15507.7 (±15507.7) | 1461.4 (±658.8) | 0.095 (± 0.048) |
| Hum (n=30) | 266.1 (±72.2) | 262.4 (±69.1) | 1.214 (±0.217) | 1.194 (±0.261) | 0.543 (±0.290) | 130.3 (±58.5) | 4926.2 (±4926.2) | 617.2 (±684.2) | 0.204 (± 0.191) |
| Hum gradation (n=29) | 998.9 (±1589.6) | 1958.8 (±2782.5) | 2.207 (±0.916) | 1.547 (±0.770) | 1.105 (±0.445) | 98.3 (±38.6) | 10296.2 (±10296.2) | 1262.4 (±1788.6) | 0.326 (± 0.222) |
| Hum short (n=9) | 197.9 (±31.2) | 187.5 (±0) | 1.268 (±0.205) | 1.479 (±0.232) | 0.282 (±0.094) | 64.5 (±23.1) | 5828.7 (±5828.7) | 354.1 (±130.7) | 0.177 (± 0.078) |
| Isolation call (n=16) | 3237.3 (±2466.1) | 6106.2 (±2702.0) | 3.120 (±0.798) | 2.448 (±1.173) | 1.025 (±0.191) | 185.1 (±64.6) | 15063.3 (±15063.3) | 3161.1 (±2512.3) | 0.375 (± 0.202) |
| Snort (n=32) | 407.4 (±84.2) | 374.9 (±101.2) | 3.507 (±1.726) | 3.620 (±1.742) | 0.169 (±0.063) | 126.1 (±67.3) | 19950.6 (±19950.6) | 1681.6 (±1212.3) | 0.070 (± 0.039) |
| Suckling call (n=12) | 363.2 (±128.1) | 317.7 (±87.0) | 2.101 (±1.152) | 2.11 (±1.236) | 1.028 (±1.003) | 135.9 (±44.4) | 12710.8 (±12710.8) | 1703.1 (±1962.7) | 0.725 (± 1.022) |
| Underwater call (n=9) | 203.1 (±70.3) | 213.5 (±99.7) | 1.316 (±0.077) | 1.310 (±0.069) | 0.746 (±0.379) | 80.0 (±32.5) | 655.4 (±655.4) | 203.1 (±70.3) | 0.269 (± 0.183) |
| Wavering scream (n=11) | 607.2 (±428.6) | 822.6 (±1197.2) | 1.574 (±0.336) | 1.522 (±0.671) | 1.376 (±0.690) | 181.8 (±68.8) | 6533.8 (±6533.8) | 1397.7 (±378.3) | 0.896 (± 0.691) |
| Whine (n=20) | 387.4 (±75.6) | 375.7 (±53.7) | 1.282 (±0.051) | 1.294 (±0.081) | 0.695 (±0.354) | 179.2 (±22.6) | 10386.2 (±10386.2) | 1675.7 (±535.9) | 0.376 (± 0.247) |
| Whistle (n=18) | 5749.9 (±1526.6) | 6700.5 (±1365.2) | 2.076 (±0.624) | 2.091 (±0.636) | 0.196 (±0.055) | 2553.2 (±788.5) | 17112.0 (±17112.0) | 5963.5 (±1556.5) | 0.098 (± 0.056) |
| Whistle double (n=13) | 7427.8 (±1400.0) | 7427.8 (±1597.9) | 2.240 (±0.541) | 2.309 (±0.408) | 0.489 (±0.203) | 2701.6 (±1689.6) | 20795.6 (±20795.6) | 6822.1 (±1929.7) | 0.217 (± 0.121) |
