# Supplementary material for: The Vocal Repertoire of Adult and Neonate Giant Otters (Pteronura brasiliensis)
Source: PLoS One. 2014 Nov 12;9(11):e112562. doi: 10.1371/journal.pone.0112562 (PMC4229255; doi:10.1371/journal.pone.0112562)
Supplement: Table S2 — Comparison of social organization and vocal repertoire size in eight of the 13 otter species. (DOCX) [file pone.0112562.s002.docx]

Table S2: Comparison of social organization and vocal repertoire size in eight of the 13 otter species.

| **Species** | **Social organization** | **Group size (nr of individuals)** | **Number of distinct vocalizations** |
| --- | --- | --- | --- |
| *Lontra longicaudis* | Solitary*, solitary foraging [41,83] | 1* [41] | > 4 [83] |
| *Lutra lutra* | Semi-solitary [86], solitary foraging [41] | 1* [41] | 8 [79] plus gradations [80] |
| *Aonyx capensis* | Semi-solitary [55], sometimes seen in groups of 8 individuals, mother-offspring or male groups, occasionally food-sharing but solitary foraging [41] | 1-8 [41] | ~ 13[55] |
| *Enhydra lutris* | Semi-solitary with gregarious female and male groups, solitary foraging [41] | 70-2000 males [41,88,89] 2–5 females [41] | 10 plus gradations [81] |
| *Lontra canadensis* | Semi-solitary with males being more gregarious, cooperative foraging [87] | 1-30 [41] | 11 [85] |
| *Aonyx cinerea* | Social, groups include both parents with offspring and helpers [41,91-93] | 4-15 [41] | 7 plus gradations [82], 12 [84] |
| *Lutrogale perspicillata* | Social, family groups with or without adult male [41,56] or groups of 3-11 individuals [41,90], cooperative foraging [41,56] | 3-11 [41] | >5 [56] |
| *Pteronura brasiliensis* | Social, groups include both parents with offspring and helpers [42], group foraging [41,42] | 3-20 [41,42] | 9 [42], 19 [54], 22 in this study |

*Solitary: individuals live solitary most of the year, and we included groups of mother and offspring of the current year as solitary living.
